# 

**Published:** 1904-02-06

**Authors:** 


### CONTENTS.

**Municipal Milk Supply** - 327
**The Teaching of Hygiene and Temperance to School Children** - 328
**Annotations** - 329
**Notes and News** - 330
**Hospital Clinics and Medical Progress—**
What We Owe to Experiments on Animals - 331
Enuresis in Children - 334
The Significance of Superficial and Tendon Reflexes in the Lower Extremity - 334
Progress of Surgery - 335
Progress in Surgery of the Brain and Nerves - 336
Progress in Gynæcology - 338
**New Appliances and Things Medical** - 338
**The Book World of Medicine and Science** - 339
**The Metric System in Medicine** - 340
**Hospital Administration—**
Hospital for Infectious Diseases, Sheffield - 341
Historical Sketch of Some of the London Hospitals - 343
Hospital Meetings - 345
The Story of the Insane from Year to Year - 346
**The Student of Medicine** - 346

*NURSING SECTION.*

**Notes on News from the Nursing World—**

Queen Alexandra's Military Nursing Service—The Anglo-American Nursing Home at Rome—Parliament and the Registration of Nurses—Irish Nurses and Registration—Nurses' Conversazione at St. Thomas's Hospital—Anglo-American Hospital, Cairo—Workhouse Infirmary Nursing Association—The Matron's Signature on Certificates—A Warning to Nurses and their Friends—Army Nurses in America—A Question of Truth—Medals for Asylum Nurses—Nurses for Emergency Cases—Victorian Trained Nurses' Association—Working Men and District Nursing—An Understaffed Workhouse Infirmary—Making a Nurse Pay for her Illness—Nurses at Play—New Nurses' Home at Salford—Short Items - 253-256

**The Nursing Outlook** - 257

**Lectures on Ophthalmic Nursing** - 258

**Royal British Nurses' Association** - 259

**Death in Our Ranks** - 259

**The Anglo-American Nursing Home at Rome** - 260

**Presentations** - 261

**Where to Go** - 261

**Everybody's Opinion** - 261

**Appointments** - 262

**Echoes from the Outside World** - 263

**Notes and Queries** - 264

**For Reading to the Sick** - 264

